# Glaucoma and Autoimmunity: Immunopathogenic Mechanisms and Emerging Immunomodulatory Therapies

**DOI:** 10.3390/biomedicines14061209

**Published:** 2026-05-27

**Authors:** Murong Wang, Chunying Liu, Xin Wei

**Affiliations:** Department of Ophthalmology, West China Hospital of Sichuan University, Chengdu 610000, China; wangmurong311@163.com (M.W.); treasure1708@163.com (C.L.)

**Keywords:** glaucoma, autoimmunity, immune dysregulation, neuroinflammation, retinal ganglion cells, immunomodulatory therapy

## Abstract

Glaucoma is a chronic progressive optic neuropathy and one of the leading causes of irreversible blindness worldwide. Although elevated intraocular pressure remains the most important modifiable risk factor, increasing evidence suggests that immune dysregulation and autoimmune responses also contribute substantially to disease onset and progression. Clinical studies across different glaucoma subtypes have identified subtype-dependent immune abnormalities, including altered serum autoantibody profiles, dysregulated cytokine and chemokine expression, and changes in peripheral immune cell subsets. Experimental and translational studies further indicate that multiple immunopathogenic mechanisms are involved in glaucomatous neurodegeneration, including glial cell-mediated immune responses, activation of pattern recognition receptor signalling pathways, adaptive immune responses, and complement cascade dysregulation. These processes may interact to sustain chronic neuroinflammation, promote retinal ganglion cell injury, and accelerate optic nerve degeneration. Importantly, a better understanding of immune involvement in glaucoma has generated growing interest in immunomodulatory therapy as a potential strategy beyond intraocular pressure lowering. Targeting microglial activation, inflammatory signalling pathways, adaptive immune imbalance, and complement-mediated injury has shown neuroprotective potential in animal or in vitro models, whereas clinical evidence in glaucoma patients remains limited. These findings may provide preliminary directions for future therapeutic development. In this review, we summarise the current clinical evidence linking glaucoma with autoimmunity, discuss the major immune mechanisms implicated in disease pathogenesis, and highlight recent advances in immunomodulatory therapeutic strategies. Elucidating the immune basis of glaucoma may help pave the way for more precise and effective treatments for this complex optic neuropathy. We believe that immune dysregulation in glaucoma functions as a context-dependent amplifier of retinal ganglion cell injury rather than a uniform primary driver, with innate (microglia/astrocytes), adaptive (T/B cells, HSP-specific immunity), and complement pathways interacting to sustain neuroinflammation and neurodegeneration. This integrated immune response contributes to subtype- and stage-specific vulnerability, and targeting these maladaptive immune mechanisms represents a promising, precision-guided strategy for neuroprotection beyond intraocular pressure lowering.

## 1. Introduction

Glaucoma is a chronic optic neuropathy characterised primarily by progressive degeneration of retinal ganglion cells (RGCs) and their axons. Retinal ganglion cells, the final output neurons of the retina whose axons form the optic nerve and transmit integrated visual information from the inner retina to the brain, are the principal neuronal population damaged in glaucoma. Glaucoma ranks among the leading causes of irreversible blindness globally, typically presenting with elevated intraocular pressure (IOP), defined as the pressure applied to the ocular wall by intraocular structures and fluids, together with visual field defects and progressive enlargement of the cup-to-disc ratio [1]. Advanced age and elevated IOP are established risk factors, and the global prevalence of glaucoma is projected to reach 111.8 million by 2040 [2].

Elevated IOP can damage RGCs by increasing mechanical load on the optic nerve head and impairing axonal transport, but this does not fully explain all glaucoma subtypes. In normal-tension glaucoma (NTG), a form of open-angle glaucomatous optic neuropathy characterised by optic disc/RNFL damage and corresponding visual field loss despite IOP remaining within the statistically normal range, indicating additional non-IOP-dependent pathogenic mechanisms. Current clinical diagnosis and treatment of glaucoma remain primarily centred on elevated IOP, with pressure reduction being the only intervention evidence-based medicine has demonstrated to delay disease progression [3,4]. Clinical management remains focused on lowering IOP, yet many patients continue to experience visual deterioration, underscoring the importance of other pathological processes.

RGC death involves stress responses, including sustained inflammatory signalling, which may represent secondary inflammatory responses to IOP-induced or neurodegenerative stress, with emerging evidence implicating an autoimmune process in glaucoma pathogenesis, with altered antibody profiles, immune cell dysfunction, and local inflammation observed in patients [5]. Consequently, investigating the relationship between glaucoma and autoimmunity not only deepens our understanding of the disease’s pathogenesis but may also provide theoretical foundations for early diagnosis, subtype classification, and immunomodulatory therapeutic strategies.

## 2. Clinical Immunological Evidence in Glaucoma Patients

### 2.1. Autoimmune Comorbidity

Approximately 30% of patients with normal-tension glaucoma have been reported to present with concomitant autoimmune diseases [6], among which rheumatoid arthritis appears particularly common. Notably, several shared autoantigens exist between these conditions and glaucoma [7]. Compared to controls, patients with primary open-angle glaucoma (POAG) exhibit higher prevalence rates of autoimmune disorders, including rheumatoid arthritis, psoriasis, and non-infectious anterior uveitis [8]. Similarly, patients with systemic autoimmune diseases appear to have a significantly elevated risk of developing glaucoma. The risk of POAG is highest within two years of rheumatoid arthritis diagnosis, with an increased risk observed in patients aged 75 years and above [9]. Patients with vitiligo exhibit an elevated risk of glaucoma, with a higher risk specifically for POAG [10]. Patients with systemic autoimmune diseases, including Stevens-Johnson syndrome, develop new-onset glaucoma more frequently and progress faster after Boston I-type KPro corneal implantation than those with other preoperative diagnoses [11]. These epidemiological and comorbidity data provide clinically relevant evidence that glaucoma, particularly NTG and POAG, may occur more frequently in systemic immune-dysregulated contexts. However, the strength of this evidence should be interpreted cautiously. Most studies in this category are case–control, retrospective cohort, or population-based association studies, which are valuable for detecting disease-level associations but cannot by themselves establish causality. Several confounders may contribute to the observed associations, including age, vascular comorbidity, corticosteroid exposure, ocular inflammation, treatment history, and surveillance bias among patients with chronic systemic diseases who undergo more frequent ophthalmic evaluation. Therefore, autoimmune comorbidity should not be interpreted as direct proof that autoimmunity initiates glaucoma in all patients. Rather, these findings support the concept that systemic immune dysregulation may define a susceptible subgroup in which immune-mediated vascular dysfunction, inflammatory priming, or altered neuroimmune responses may lower the threshold for glaucomatous optic neuropathy.

### 2.2. Autoantibody Profiles and Cytokine Changes

Humoral immune alterations have been observed across glaucoma subtypes. POAG patients exhibit elevated autoantibodies against HSP27, HSP60, CALD1, PGAM1, VDAC2, and HSPD1, with some antibodies correlating with disease severity and appearing even in early-stage disease [12]. β2-adrenergic receptor agonistic autoantibodies (β2-agAAb) are present in 82% of POAG patients, absent in healthy controls [13], and may influence IOP regulation and retinal microcirculation [14]. Additionally, acute IOP elevations can induce transient remodelling of autoantibody profiles, such as anti-HSP27, anti-TTLL12, and anti-NSE, indicating dynamic immune responsiveness [15]. Beyond humoral immunity, some glaucoma patients show systemic immune activation or dysregulation, as reflected by peripheral blood lymphocyte gene expression, T-cell subset distribution and activity, and Th1/Th2 or other cytokine axis shifts [16,17]. Peripheral immune dysregulation in glaucoma should not be interpreted as a straightforward Th1-to-Th2 shift, but rather as a multilayered immune imbalance involving IL-4/IL-6 elevation, antigen-induced Th2-like deviation, impaired regulatory T-cell control, and enhanced pro-inflammatory responsiveness [18,19,20]. These findings suggest potential biomarker roles, but whether these humoral changes are pathogenic drivers or stress-response indicators remains uncertain.

### 2.3. Peripheral Immune Cell Remodelling and Complement Activation

Peripheral immune cell analyses reveal significant alterations in glaucoma patients’ T-cell subsets, with reduced CD4^+^CD25^+^FoxP3^+^ Tregs, decreased Treg/Th1 and Treg/Th17 ratios, and enhanced cytokine release upon stimulation [19]. Patients with POAG exhibit marked immune cell remodelling, characterised by increased proportions of CD4^+^ T lymphocytes and myeloid cells, alongside reduced proportions of terminally differentiated CD8^+^ GZMK^+^ T cells and natural killer cells, resulting in impaired cytotoxic potential [21]. At the tissue level, retinal complement components C1q, C3, and membrane attack complex (MAC) show increased deposition in glaucomatous eyes, alongside reduced CFH expression, suggesting maladaptive complement activation contributing to RGC apoptosis [22,23].

Table 1 systematically summarises clinical immunological manifestations and relevant evidence in glaucoma patients, covering autoimmune comorbidities, autoantibody characteristics, cytokine changes, peripheral immune cell remodelling, and complement activation.

### 2.4. Integrative Interpretation

Taken together, clinical evidence supports an immune-associated glaucoma phenotype encompassing autoimmune comorbidity, altered autoantibody profiles, cytokine network remodelling, peripheral immune cell dysregulation, and local complement activation. The strongest evidence applies to NTG and POAG subgroups with systemic immune dysregulation. Nevertheless, most data remain associative, heterogeneous, and influenced by disease stage, assay methodology, and patient stratification. Current evidence should be interpreted as indicating disease modification potential rather than universal causation. Future longitudinal, subtype-specific, and mechanistically anchored studies are required to clarify whether these immune abnormalities act as pathogenic drivers, compensatory responses, or secondary consequences of chronic glaucomatous neurodegeneration.

## 3. Immune Mechanisms

### 3.1. Innate Immunity

#### 3.1.1. Microglia-Mediated Immune Response

Microglia are highly specialised tissue-resident macrophages located within the CNS parenchyma, distinct from CNS-associated macrophages residing at interfaces such as the leptomeninges, perivascular spaces, and choroid plexus [24]. They arise from yolk-sac erythro-myeloid progenitors during embryogenesis and are maintained as a long-lived, locally self-renewing macrophage population within the CNS [24,25]. In the healthy brain, microglia remain active, continuously surveying the parenchyma with highly motile processes and rapidly responding to local tissue injury, as revealed by in vivo two-photon imaging [26]. Beyond surveillance, microglia contribute to neural circuit refinement by engulfing presynaptic inputs during postnatal synaptic pruning through neuronal activity-dependent and CR3/C3 complement-dependent mechanisms [27]. Under pathological conditions, microglia can acquire reactive phenotypes involving morphological remodelling, enhanced phagocytosis, cytokine and chemokine production, complement-related responses, oxidative mediator release, and antigen-presenting functions [24]. These responses may support host defence, debris clearance, and tissue repair, whereas sustained or dysregulated activation may contribute to neuroinflammation and neurodegenerative pathology; in particular, complement- and microglia-dependent synapse elimination has been implicated in disease-associated synaptic loss [24,28,29]. Microglia activation constitutes a pivotal pathological event in the early stages of glaucoma. Within the DBA/2J glaucoma mouse model, microglia exhibit activation before RGC loss, manifested through increased Iba1 expression, cellular aggregation, and morphological transformation. Moreover, the degree of activation within the optic nerve head (ONH), the posterior ocular region where retinal ganglion cell axons converge, traverse the lamina cribrosa, and exit the eye to form the optic nerve, correlates significantly with the severity of subsequent neural damage [30,31]. In experimental models of elevated IOP, increased ocular pressure activates microglia via the mechanosensitive channel PIEZO1, triggering the release of pro-inflammatory mediators [32,33]. Microglia express the mechanosensitive ion channel PIEZO1, which is enriched in brain endothelial cells and microglia, providing a molecular basis for their ability to sense changes in tissue stiffness and membrane tension. Persistent IOP elevation imposes chronic strain on the optic nerve head and retinal neural tissues, leading to repeated activation of PIEZO1, Ca^2+^ influx, and downstream inflammatory programs such as HIF1α-related signalling. Meanwhile, pressure-induced axonal and RGC injury generates damage-associated molecular cues and cellular debris, which further reinforce microglial phagocytic and inflammatory phenotypes. Activated microglia participate in the pathological process through dual mechanisms: initially exerting neuroprotective effects by phagocytosing apoptotic cells and secreting brain-derived neurotrophic factor [34], whilst sustained activation releases pro-inflammatory factors such as TNF-α and IL-1β alongside reactive oxygen species, directly inducing RGC apoptosis [35]. Furthermore, microglia can detect extracellular DNA via the cGAS-STING pathway, amplifying the inflammatory response and exacerbating retinal damage [36].

The interactive network between microglia and other cells is pivotal in amplifying glaucomatous inflammation. Activated microglia secrete factors such as C1q and IL-1β, inducing astrocytes to transform into a neurotoxic A1 phenotype and disrupting the blood-retinal barrier (BRB), a highly selective vascular interface formed by retinal endothelial cells, pericytes, Müller glia, astrocytes, and tight junction complexes that maintains retinal immune privilege and regulates the exchange of fluids, solutes, immune cells, and inflammatory mediators between the circulation and the neural retina [34,37]. Müller cells release ATP via connexin-43 hemichannels, activating P2X7 receptors on microglial surfaces to promote their proliferation and migration [38,39]. In normal-tension glaucoma models, microglia reduce retinal vascular density by regulating the rpl17/stat5b/apoa1 axis, indirectly exacerbating RGC injury [40]. Monocyte infiltration synergises with microglial activation; CCL2-CCR2 pathway-mediated monocyte recruitment further amplifies inflammatory responses [34].

**Figure 1:**
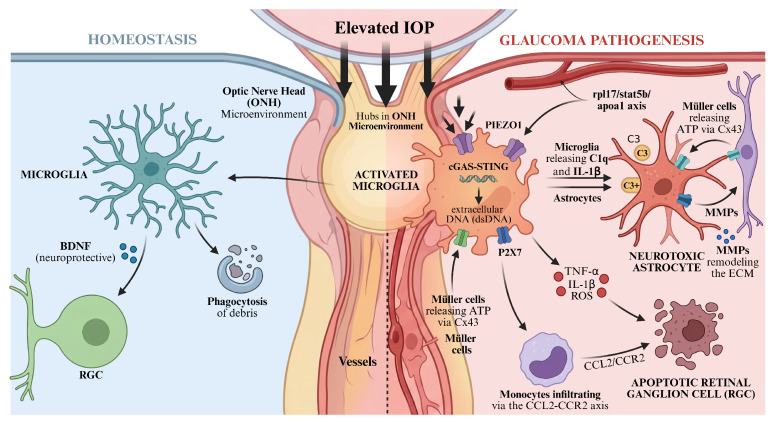
Mechanistic Schematic of Glial-Mediated Neuroinflammation in the Optic Nerve Head During Glaucoma Pathogenesis: Elevated IOP activates microglia, Müller cells, and astrocytes, triggering mechanosensitive signalling, ATP release, cGAS–STING activation, complement cascade, and chemokine-mediated monocyte infiltration. Together, these interconnected pathways amplify neuroinflammation, promote extracellular matrix remodelling, and drive the apoptosis of retinal ganglion cells, ultimately contributing to progressive glaucomatous optic nerve damage.

The immunophenotypic plasticity of astrocytes plays a dual role in glaucoma pathology: their excessive activation induces immune dysregulation closely associated with RGC apoptosis [41], whereas moderate immune responses may exert neuroprotective effects by clearing damaged products [42]. Elevated IOP induces ONH astrocytes to adopt a neurotoxic (A1) phenotype, promoting a neuroinflammatory environment via increased complement C3 and inflammatory cytokine expression [43]. Hernandez et al. identified abnormal expression of connexin 43 (Cx43) in ONH astrocytes of glaucoma patients, disrupting gap junction communication and further promoting pro-inflammatory factor release [44]. Concurrently, astrocytes remodel the extracellular matrix by secreting matrix metalloproteinases, thereby destabilising the optic nerve microenvironment [44] (Figure 1).

#### 3.1.2. Activation of Pattern Recognition Receptor Signalling Pathways

Proteomic and immunohistochemical studies of human glaucomatous retinal tissue confirm that TLR2, TLR3, TLR4, and other receptors exhibit significantly upregulated expression, primarily localised within retinal microglia and astrocytes [45]. Elevated heat shock proteins (HSPs) and oxidative stress products within the glaucomatous tissue microenvironment activate glial cells via TLR signalling pathways. This promotes the release of pro-inflammatory factors (such as TNF-α) and the expression of MHC class II molecules, thereby enhancing antigen presentation capacity and stimulating T-cell proliferation. This process initiates a cascade of activation linking innate and adaptive immune responses [45].

**Figure 2:**
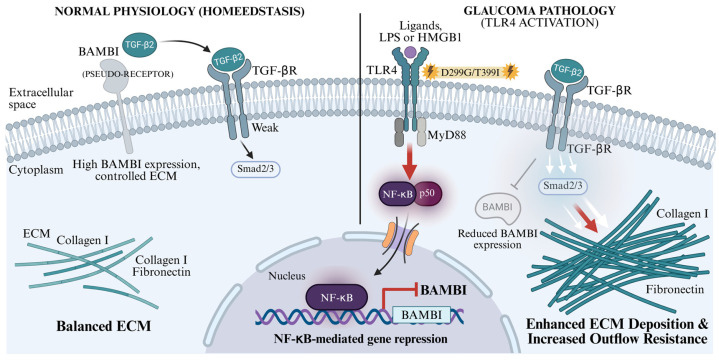
Mechanistic Schematic of TGF-β2/BAMBI/NF-κB Axis in Trabecular Meshwork Extracellular Matrix Remodelling During Glaucoma: TLR4 activation in trabecular meshwork cells suppresses BAMBI, enhances TGF-β/Smad signaling, promotes collagen and fibronectin deposition, and increases aqueous outflow resistance, representing a POAG-relevant mechanism of IOP elevation.

TLR4 appears particularly relevant to POAG and IOP-dependent glaucomatous mechanisms. In trabecular meshwork cells, TLR4 activation may suppress BAMBI through the MyD88/NF-κB pathway, thereby enhancing TGF-β2-driven extracellular matrix deposition, increasing aqueous outflow resistance, and contributing to IOP elevation. Whether the same pathway is equally relevant to NTG remains insufficiently established [46]. Concurrently, TLR4 binds to damage-associated molecular patterns in aqueous and vitreous fluids (e.g., HMGB1, HSP72), mediating RGC apoptosis via the NLRP3 inflammasome and caspase-8 pathways. Inhibiting TLR4 significantly enhances RGC survival [46]. Genetic polymorphism analysis revealed increased frequencies of the TLR2-753 ArgArg and TLR6-249 ProPro genotype combinations in POAG patients. Linkage disequilibrium exists within the TLR2-TLR6 and TLR4 genes, suggesting intergenic interactions may influence disease susceptibility by altering receptor-ligand binding capacity or signalling efficiency [47]. This genetic background variation may account for the heterogeneity in POAG risk across different populations [47] (Figure 2).

### 3.2. Adaptive Immunity

In a healthy state, the eye possesses immune privilege, with the retina restricting the entry of peripheral immune cells via the BRB and immunoregulatory mechanisms to prevent inflammatory damage [48,49]. However, stress signals in glaucoma—such as elevated IOP, oxidative stress, haemodynamic abnormalities, and microglial activation—induce increased BRB permeability, thereby triggering alterations in the local immune microenvironment [50]. Studies indicate that T-cell-deficient mice do not exhibit progressive RGC loss following elevated IOP, whereas transferring CD4^+^ T cells from glaucoma model mice into T-cell-deficient mice reproduces the RGC injury phenotype [51,52]. However, these findings mainly reflect defined animal models of IOP-induced injury rather than direct proof of a universal mechanism in human glaucoma. HSPs, as highly conserved stress proteins, serve as key autoantigens in the adaptive immune response to glaucoma [53]. An abnormal peripheral T-cell response to HSP-like autoantigens may represent an important mechanism in selected glaucoma contexts, particularly in experimental IOP-elevation models and in POAG/NTG cohorts showing enhanced HSP-specific immune reactivity. Elevated IOP induces upregulation of HSP27 and HSP60 expression in RGCs and retinal glial cells, with these proteins subsequently released extracellularly to serve as danger signals activating T cells [54]. In glaucoma patients, the frequency of HSP27- and HSP60-specific T cells in peripheral blood is more than fivefold higher than in healthy individuals, with corresponding autoantibody levels in serum also significantly elevated [7]. Transient elevation of intraocular pressure in animal models has been shown to induce CD4^+^ T-cell infiltration into the retina. This infiltration persists long after pressure normalisation, with these T-cells specifically recognising HSP27 and HSP60 antigens. This response is markedly absent in germ-free mice [51]. Nevertheless, HSP-specific immunity may represent either a pathogenic driver or a secondary response to retinal stress, and its contribution is likely to vary by glaucoma subtype, disease stage, and systemic immune background.

The gut-retina axis plays a pivotal role in adaptive immune activation during glaucoma [55]. Alterations in gut microbiota composition have been reported in glaucoma-related studies, whereas the association between Helicobacter pylori infection and disease risk appears most clearly linked to POAG. Therefore, gut–retina immune interactions should be discussed as a potential, but not yet universally established, mechanism across glaucoma subtypes. In contrast, human studies mainly report associative changes in gut microbiota composition in POAG, which may be influenced by age, diet, geography, medication use, and systemic comorbidities. HSPs derived from gut microbiota activate peripheral β7^+^CD4^+^ T cells, which infiltrate the retina by traversing the BRB through binding to MAdCAM-1 on retinal vascular endothelial cells. He et al. demonstrated that neutralising MAdCAM-1 significantly reduced T-cell infiltration and mitigated RGC damage, suggesting that gut microbiota-sensitised T-cells constitute key effector cells in the autoimmune response of glaucoma.

The interaction between adaptive and innate immunity further amplifies neurotoxic effects [56]. Infiltrating CD4^+^ T cells activate retinal microglia by secreting IFN-γ, inducing an M1 phenotype and prompting the release of pro-inflammatory factors such as TNF-α and IL-1β, thereby establishing an inflammatory amplification loop [56]. Concurrently, activated microglia, acting as antigen-presenting cells, further stimulate T cells via MHC class II molecules, perpetuating chronic inflammation [56]. This sustained immune activation induces RGC apoptosis via the Fas-FasL pathway, which may progress even after IOP normalises [51,55]. These findings are mainly observed in IOP-elevation animal models or limited patient cohorts, and may not universally represent human glaucoma pathogenesis.

### 3.3. Complement System

The complement system, as a core component of innate immunity, comprises multiple plasma proteins that mediate pathogen opsonisation, inflammatory amplification, and effector cell lysis through a cascade reaction. It simultaneously serves as a bridge between innate and adaptive immunity. Complement proteins predominantly exist in zymogen form, primarily activated via the classical pathway, lectin pathway, and alternative pathway, ultimately forming the MAC. This complex disrupts the target cell membrane integrity and induces cell lysis [57]. Importantly, complement activity in the nervous system is not intrinsically pathological. During retinal and CNS development, C1q and C3 participate in activity-dependent synaptic pruning by tagging weak or redundant synapses for microglial elimination, thereby contributing to circuit refinement. In the adult retina, low-level complement activity may also support tissue homeostasis by facilitating the clearance of apoptotic cells, cellular debris, and damaged synaptic elements [58,59,60].

**Figure 3:**
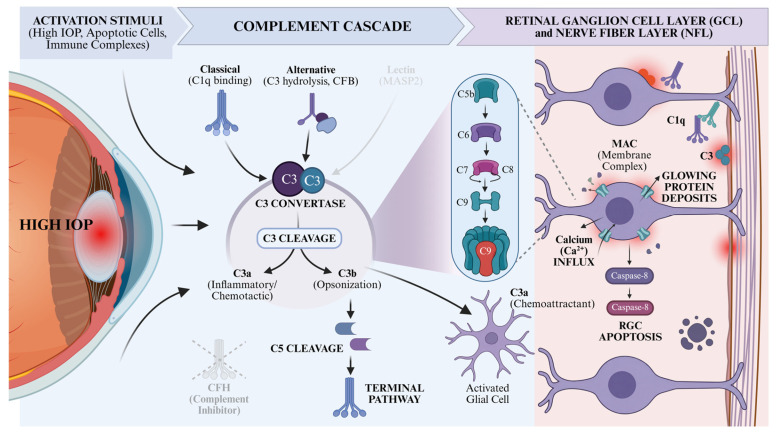
Complement Cascade Activation in Response to Elevated Intraocular Pressure Drives Retinal Ganglion Cell Apoptosis in Glaucoma: Elevated IOP and immune-related stress activate the complement cascade, leading to C3 cleavage, glial activation, membrane attack complex formation, calcium influx, and retinal ganglion cell apoptosis in glaucoma.

While clinical tissue studies confirm deposition, distinguishing primary pathogenic activity from secondary neurodegenerative responses remains challenging. Clinical studies have confirmed that glaucoma patients exhibit increased deposition of complement components C1q, C3, and MAC in the retina and aqueous humour, alongside downregulated expression of the complement inhibitor CFH (complement factor H). Furthermore, the C3a/C3 ratio in the aqueous humour and serum of advanced-stage patients is significantly elevated, correlating closely with disease progression rate [22,61,62]. Animal studies further validate that elevated IOP induces upregulation of C1qb and C3 gene expression in rat retinas. Their products specifically deposit within the ganglion cell layer and nerve fibre layer, ultimately forming MAC that triggers RGC apoptosis [23,63]. The complement system participates in glaucoma pathogenesis via three pathways: the classical pathway is activated by apoptotic cells or immune complexes, with C1q binding initiating the cascade reaction [64]; the alternative pathway undergoes sustained activation through spontaneous C3 self-cleavage, with its key component CFB exhibiting elevated expression in glaucoma models; CFB knockout significantly reduces RGC loss [65]; although MASP2 upregulation has been detected in the complement-binding pathway in some models, its activation remains limited in high IOP models [61,66]. Ultimately, all three pathways generate C3a (chemoattractant for inflammatory cells) and C3b (complement-opsonisation) via C3 cleavage, forming the MAC to disrupt cell membrane integrity. Concurrently, they activate intrinsic and extrinsic apoptosis pathways through calcium influx and caspase-8/caspase-9 activation [67]. Furthermore, complement activation induces glial cell activation, releasing pro-inflammatory mediators that exacerbate neuroinflammation [23,63] (Figure 3).

Therefore, complement becomes maladaptive in glaucoma when a normally protective clearance program is chronically reactivated or insufficiently restrained by complement regulators. Under sustained IOP-related stress, oxidative injury, mitochondrial damage, glial activation, or blood-retinal barrier disruption, complement tagging may extend from damaged debris to stressed but still viable RGC synapses, dendrites, and somata, thereby converting homeostatic clearance into pathological synapse loss, inflammatory amplification, and neuronal injury [68,69]. The temporal role of complement is also critical. Experimental glaucoma studies suggest that C1q- or C3-mediated synaptic tagging and microglial pruning may occur early, before overt RGC loss, whereas robust C3 activation, MAC deposition, and apoptosis-related signalling may become more prominent during progressive or advanced disease. Complement inhibition may be most effective when applied early to mid-stage, while maladaptive synaptic pruning and inflammatory amplification are active, but irreversible neuronal loss remains incomplete. Conversely, broad or late-stage complement blockade may be less effective and could interfere with physiological debris clearance and tissue repair [68].

Table 2 illustrates integrated immune mechanisms and therapeutic targets during glaucoma progression. This table summarizes pivotal innate, adaptive and complement immune mechanisms of glaucoma, as well as relevant therapeutic targets.

## 4. Potential Therapeutic Strategies Based on Immunomodulation

### 4.1. Targeting the Innate Immunity

#### 4.1.1. TNF Inhibitor

Etanercept, as a TNF inhibitor, competitively binds to circulating TNF-α and blocks its interaction with cell surface receptors, thereby suppressing downstream inflammatory signalling. It has been extensively employed in the treatment of various inflammatory immune disorders, including rheumatoid arthritis, psoriatic arthritis, juvenile idiopathic arthritis, and ankylosing spondylitis [70,71]. Roh et al. demonstrated in a rat glaucoma model that immunofluorescence confocal imaging revealed TNF-α under elevated IOP primarily originates from microglia surrounding the ONH [72]. Etanercept significantly reduced the number of activated microglia in the ONH region and inhibited their transition from a resting dendritic morphology to an amoeboid activated state. Structurally and functionally, elevated IOP caused a 40.2% reduction in optic nerve axon density, accompanied by axonal swelling, demyelination, and vacuolation. Etanercept maintained axon density at near-normal levels and preserved essentially normal optic nerve axons. After 14 days of elevated IOP, retinal nerve fibre light chain and medium chain (NF-L/NF-M) expression decreased, whereas etanercept maintained levels close to normal. Four weeks of elevated IOP caused a 38% reduction in RGCs, which etanercept prevented, maintaining cell density close to that of the sham group. Notably, intraperitoneal injection of etanercept did not affect persistently elevated IOP, suggesting its action is independent of IOP reduction [72]. Thus, in experimental IOP-dependent glaucomatous injury, TNF-α inhibition by etanercept mitigates axonal degeneration and protects RGCs without lowering IOP. These findings support TNF-α blockade as a precision immunotherapy approach for POAG patients with pronounced inflammation or systemic autoimmune comorbidity. Its application in NTG or non-IOP-dependent disease should be guided by biomarker evidence of microglial activation or TNF-driven immune dysregulation. However, further research is required to address issues such as long-term safety concerns, ensuring effective drug delivery to the optic disc, sustained efficacy, and synergistic effects with existing IOP-lowering medications.

Adalimumab, a fully human monoclonal antibody against TNF-α, has not yet been supported by direct evidence as a standard therapy for primary glaucoma. Nevertheless, the TNF-α receptor axis targeted by adalimumab has been implicated in several key mechanisms of glaucomatous neurodegeneration, including glial activation, retinal ganglion cell (RGC) apoptosis, axonal degeneration, and secondary neurodegenerative injury [73,74]. Adalimumab has demonstrated robust anti-inflammatory and corticosteroid-sparing effects in non-infectious uveitis and has accumulated substantial clinical experience in ophthalmic inflammatory diseases. In the randomised, double-masked, placebo-controlled phase III VISUAL II trial, adalimumab significantly reduced the risk of treatment failure related to uveitic flare or visual acuity loss during corticosteroid withdrawal in patients with inactive non-infectious uveitis [75,76]. Therefore, adalimumab may be considered a candidate immunomodulatory therapy worthy of further investigation in glaucoma, particularly in patient subgroups characterised by prominent intraocular inflammation, autoimmune comorbidity, or uveitic secondary glaucoma. However, given the context-dependent and bidirectional roles of TNF-α/TNFR1/TNFR2 signalling, complete TNF-α blockade may also interfere with TNFR2-mediated neuroprotective and tissue-reparative pathways. Future studies should therefore define the most appropriate target population, route of administration, therapeutic window, and receptor-selective strategies for modulating TNF signalling in glaucomatous neurodegeneration [77,78]. Although adalimumab has demonstrated anti-inflammatory effects in uveitis, its relevance to primary glaucoma is speculative. Complete TNF-α blockade may interfere with neuroprotective TNFR2 pathways. Careful determination of target populations, therapeutic windows, and receptor-specific modulation is needed before clinical translation.

#### 4.1.2. Bupropion

Bupropion is a noradrenaline-dopamine reuptake inhibitor, commonly used clinically as an antidepressant and also effective in smoking cessation [79]. Brustolim and colleagues hypothesised that this drug inhibits TNF synthesis by increasing extracellular noradrenaline and dopamine, thereby elevating intracellular cyclic adenosine monophosphate (cAMP) levels [80]. Animal studies have also demonstrated that bupropion reduces serum TNF levels [81]. Stein et al. conducted a retrospective clinical study to investigate the association between bupropion use and the risk of developing open-angle glaucoma (OAG). Results indicated that bupropion use was associated with a reduced risk of new-onset OAG, showing a trend of mild risk reduction with increasing duration of use. Epidemiological data suggest long-term bupropion use may reduce OAG risk; however, this represents an association and does not establish a validated therapeutic effect. Epidemiological data suggest that long-term bupropion use is associated with a reduced risk of developing OAG, though causal mechanisms remain uncertain and the observed effect may not be fully explained by TNF inhibition [82]. This observation should be interpreted as an epidemiological association rather than a validated therapeutic strategy. However, researchers noted that this effect may not be entirely explained by TNF inhibition and could also relate to potential benefits of cAMP signalling on trabecular meshwork and RGC function [83,84]. Clinically, this suggests bupropion’s overall adverse reaction profile is relatively manageable, though its increased risk of seizures warrants caution [82]. Further rigorous clinical studies are required to validate causal pathways and determine optimal dosing windows. Epidemiological data suggest long-term bupropion use may reduce OAG risk; however, this represents an association and does not establish a validated therapeutic effect.

#### 4.1.3. Minocycline

Minocycline, as a tetracycline derivative capable of traversing the BRB, possesses anti-inflammatory, anti-apoptotic, and immunomodulatory properties [85,86,87]. Extensive animal studies have demonstrated that it may offer novel therapeutic strategies for glaucoma by regulating glial cell activation, inhibiting inflammatory pathways, and mitigating immune-mediated damage [35,88,89]. Abnormal glial cell activation lies at the core of optic nerve inflammation in glaucoma, with excessive microglial activation releasing pro-inflammatory factors that exacerbate neural damage [35,90]. In the DBA/2J glaucoma mouse model, long-term minocycline intervention reduced retinal microglial activation levels, maintained their quiescent morphology, and downregulated expression of the activation marker Iba1 [88]. Minocycline exhibits selective regulation of microglia, preferentially inhibiting MHCII+ inflammatory leukocyte aggregation without affecting retinal resident microglial numbers. This preserves glial physiological function while suppressing pathological inflammation [91].

In laser-induced rat glaucoma and optic nerve transection models, minocycline treatment increased RGC survival rates and significantly delayed apoptosis [86]. Its protective mechanism correlates with upregulation of anti-apoptotic proteins Bcl-2, XIAP, and survivin expression. In human trabecular meshwork cells and optic nerve head astrocytes, it counteracts oxidative stress and TGFβ2-induced apoptosis [92]. Minocycline may serve as a precision immunomodulator for NTG patients or POAG patients with progressive neuroinflammation despite IOP control, with treatment guided by biomarkers such as microglial activation markers (Iba1/CD68) and pro-inflammatory cytokine levels [86,88]. However, limitations remain: optimal dosage and administration routes in animal studies are unconfirmed, with high doses potentially inducing retinal toxicity [89]; long-term safety and effects on ocular immune function require further evaluation; its efficacy in advanced retinal neurodegeneration is limited, suggesting greater suitability for early glaucoma intervention [91]. Preclinical animal studies suggest neuroprotective potential, but optimal dosing, long-term safety, and efficacy in human glaucoma remain unknown.

#### 4.1.4. Adenosine Receptor Pathway

Caffeine, an adenosine receptor antagonist, has been demonstrated to exert neuroprotective effects on the central nervous system [93,94]. Madeira et al. demonstrated in a laser photocoagulation-induced ocular hypertension (OHT) rat model that caffeine intake significantly reduced IOP in OHT rats. It also effectively suppressed retinal microglial activation, downregulated expression of microglial reactivity markers including MHC-II, TSPO, and CD11b, and reduced mRNA and protein levels of pro-inflammatory factors TNF and IL-1β [95]. Notably, caffeine also suppressed microglial reactivity in the contralateral eye unaffected by OHT. OHT upregulates retinal adenosine A_2_ₐ receptor (A_2_ₐR) expression, whereas caffeine antagonises A_2_ₐR to block microglia-mediated neuroinflammation, ultimately significantly mitigating OHT-induced RGC loss. Although caffeine failed to improve retrograde axoplasmic transport impairment in RGCs caused by OHT, it partially preserved optic nerve structural integrity. Animal studies indicate that caffeine or A_2_ₐR antagonists may mitigate microglial activation and RGC loss in OHT models. Clinical applicability remains untested, and translation to human glaucoma therapy requires further investigation [95]. In a rat model of high-pressure-induced ischaemia–reperfusion (I-R), intravitreal injection of SCH 58261 mitigated microglial activation and neuroinflammation, markedly reducing retinal cell apoptosis and RGC loss. Its neuroprotective effects were associated with modulating neuroinflammation [96]. FM101 is a highly selective A_3_ adenosine receptor (A_3_AR) modulator exhibiting both G protein-coupled receptor agonist and β-receptor antagonist activities [97]. By regulating inflammation and fibrosis-related signalling pathways, it offers a novel therapeutic approach for glaucoma. Demonstrating favourable safety profiles in acute and 28-day subchronic toxicity studies in rats, this compound has advanced to clinical trials as a potential treatment for patients with elevated IOP, evaluating its efficacy in patients with elevated IOP [98].

#### 4.1.5. Ibudilast

Ibudilast, a cyclic adenosine monophosphate (cAMP) phosphodiesterase (PDE) inhibitor with extensive clinical application, exhibits high selective affinity for PDE4 and has recently been demonstrated to possess significant neuroprotective potential [99,100]. In a rat model of elevated IOP, intravitreal injection of Ibudilast markedly suppressed activation of astrocytes and microglia within the retina and optic nerve. This reduced glial fibrillary acidic protein expression and the number of Iba1/CD68 double-positive phagocytes, thereby decreasing production of pro-inflammatory mediators such as TNF-α and IL-1β. More importantly, this drug effectively preserves RGC body survival and prevents axonal degeneration without affecting IOP, while simultaneously improving anterograde axoplasmic transport and restoring RGC signalling to the suprachiasmatic nucleus [101]. Elevated IOP induces upregulation of the PDE4A subtype in retinal Müller cells [102,103], whereas ibudilast, by inhibiting PDE4A activity, promotes cAMP accumulation within Müller cells and RGCs. This subsequently activates the protein kinase A signalling pathway, modulating glial responses, suppressing neuroinflammation, and activating protective signalling pathways, thereby offering a potential neuroprotective direction for IOP-dependent glaucomatous injury [101]. These promising preclinical results require confirmation in human glaucoma, considering appropriate dosing, ocular delivery, and long-term safety before clinical application.

#### 4.1.6. Targeting the Endothelin Axis

Howell et al. discovered in the DBA/2J glaucoma mouse model that endothelin-2 (Edn2), a potent vasoconstrictor produced by microglia/macrophages, exhibits early upregulation in the retina and optic disc head. This upregulation induces vascular dysfunction, thereby exacerbating damage to RGCs. Bosentan, a dual endothelin receptor antagonist, increases ocular blood flow in glaucoma patients without affecting blood pressure. Intervention in DBA/2J mice from 6 months of age, while not altering the progression of elevated IOP, significantly mitigated glaucomatous damage. At 10.5 months, 80% of treated mice showed no evident glaucomatous features, compared to only 39% in the control group; At 12 months, the proportion of eyes without damage remained significantly higher in the treated group [104]. Secreted Phosphoprotein 1 (SPP1), secreted by astrocytes, can be regulated via the TGF-β1/RUNX1/E2F1 pathway. It enhances mitochondrial function and phagocytosis, inhibits neurotoxicity and inflammatory cytokine production, thereby protecting RGCs. SPP1 overexpression delays age-related RGC loss and rescues visual function in glaucoma and optic nerve injury mouse models [105].

#### 4.1.7. Targeting TLR4

In the pathogenesis of POAG, TLR4, as a key receptor of innate immunity, regulates trabecular meshwork fibrosis and RGC apoptosis [46]. TLR4 gene mutation has been shown to prevent transforming growth factor-β2–induced elevation of intraocular pressure in mice. [106]; simultaneously, in optic nerve compression models, pharmacological inhibition or genetic knockout of TLR4 signalling significantly enhances RGC survival [107,108,109]. TAK242 (Resatorvid), an effective selective TLR4 inhibitor, markedly reduced RGC loss in mouse glaucoma induced by optic nerve compression [108]. In rodent studies, TAK-242 effectively inhibited the proliferation of Tenon’s capsule fibroblasts, suggesting potential application in anti-scarring therapy following glaucoma surgery [110].

#### 4.1.8. Targeting Microglia

LGALS3, as a key regulator of microglial activation [111], demonstrated significant protective effects on RGCs in both microsphere-induced and DBA/2J glaucoma models through its gene knockout or pharmacological inhibition by TD139 [112,113]. The CX3CL1-CX3CR1 signalling axis constitutes a vital inhibitory pathway for maintaining microglial homeostasis. CX3CR1 is predominantly expressed on the surface of ocular microglial cells [114]. This axis maintains microglial homeostasis by suppressing pro-inflammatory factors such as IL-1β. Intravitreal injection of recombinant CX3CL1 effectively inhibits abnormal microglial activation, demonstrating potential neuroprotective effects [115,116]. Furthermore, PLX5622-mediated microglial depletion, a CSF1R (colony-stimulating factor 1 Receptor) inhibitor, alleviates neuroinflammation and BRB disruption induced by ischaemia–reperfusion injury [117]. However, it exhibits no significant effect on acute optic nerve injury, suggesting that the therapeutic efficacy of microglia depends on disease type and timing of intervention [118].

### 4.2. Regulation of Adaptive Immunity

#### 4.2.1. Glatiramer Acetate Copolymer-1 (COP-1)

Glatiramer acetate copolymer-1 is a synthetic polypeptide composed of L-glutamic acid, L-lysine, L-alanine, and L-tyrosine. It exerts neuroprotective effects by weakly activating broadly self-reactive T cells and modulating local immune responses [119,120]. Animal studies confirm that Cop-1 immunisation significantly increases retinal T-cell accumulation in rats with elevated IOP and enhances the survival rate of RGCs [121]. Its mechanism may involve upregulating the expression of neurotrophic factors such as brain-derived neurotrophic factor (BDNF) and IGF-1, whilst balancing the secretion of pro-inflammatory factors (e.g., TNF-α) and anti-inflammatory factors (e.g., IL-10), thereby improving the retinal microenvironment [122,123]. Cop-1-activated T cells interact with microglia to reduce RGC apoptosis [122]. Preclinical animal studies suggest neuroprotective potential, but optimal dosing, long-term safety, and efficacy in human glaucoma remain unknown.

In patients with acute primary angle-closure glaucoma, Cop-1 treatment was associated with significant improvement in visual field mean deviation and fewer progressive visual field defect points compared with placebo, although RNFL thickness remained unchanged [124]. Combination therapy strategies demonstrate further advantages. Co-administration of retinal stem cell transplantation with Cop-1 immunotherapy synergistically promotes BDNF and IGF-1 secretion, further reducing RGC apoptosis rates. It also downregulates IFN-γ levels in aqueous humour and serum of glaucoma model rats, mitigating immune-mediated neuropathology [125,126]. Although Cop-1 has demonstrated favourable neuroprotective effects in animal studies and preliminary clinical trials, room for optimisation remains. Existing research suggests that variations in dosage, timing, and combination therapy regimens may influence efficacy, while their long-term safety and optimal administration route require validation through large-scale clinical studies [119,122,124,127].

#### 4.2.2. Enhancement of Tregs

By enhancing the immunoregulatory response of endogenous Tregs or employing adoptive cell therapy (such as transferring purified induced Tregs), the abnormal ocular immune microenvironment can be modulated, presenting a potential therapeutic approach for immune-mediated or inflammation-associated glaucomatous neurodegeneration [128]. Research confirms that rapamycin exerts significant protective effects on RGCs in glaucoma models [129,130]. It induces immune tolerance by suppressing the proliferation of T, B, and natural killer effector cells, while simultaneously stimulating Treg proliferation and enhancing their activity [131]. Activated Tregs are not only recruited from the bloodstream to the lesion site to suppress effector T cell activity, but may even be locally produced within the retina, further strengthening immune regulation and safeguarding ocular neural tissue function [132].

#### 4.2.3. Fas/FasL

The Fas/FasL signalling pathway occupies a pivotal position in the pathogenesis of ocular diseases, with functional differences between its subtypes and dysregulation of signalling closely associated with the progression of conditions such as glaucoma and retinal detachment [133,134,135,136,137]. Membrane-bound FasL (mFasL) exhibits pro-apoptotic and pro-inflammatory properties, whereas soluble FasL (sFasL) exerts antagonistic effects to maintain ocular immune homeostasis [134,135]. In glaucoma models, Fas receptor activation triggers RGC apoptosis, glial cell activation, and inflammatory responses [137]. Fas-deficient mice completely avoided RGC loss and axonal degeneration under high IOP conditions. The Fas receptor antagonist ONL1204, administered either before or after IOP elevation, significantly reduced RGC death and axonal damage while suppressing microglial activation and the expression of inflammatory mediators (e.g., TNF-α, IL-IL, C3) [137]. AAV-mediated sFasL gene therapy similarly achieved long-term neuroprotection in both chronic and acute glaucoma models, with mechanisms linked to the suppression of glial cell activation and inflammatory responses [134].

### 4.3. Inhibition of the Complement System

Complement system overactivation constitutes a pivotal mechanism in glaucomatous retinal neurodegeneration. Bosco et al. demonstrated that CR2-Crry retinal gene therapy can specifically inhibit complement C3 activation in glaucoma-prone mice, markedly reducing retinal C3d deposition in RGCs and inner retinal layers. This approach effectively preserves RGC soma and axonal integrity, thereby delaying optic nerve degeneration progression [138]. In a Lewis rat model of laser-induced chronic OHT, retinal C3 cleavage products and MAC levels were markedly elevated in the hypertensive eye. Intervention with the cobra venom factor CVF effectively depleted the complement system, blocked calcium influx and the upregulation of the pro-apoptotic molecule BAD, while significantly reducing MAC deposition and glial fibrillary acidic protein expression [67]. Following CVF intervention, RGC survival rates markedly increased, with reduced apoptotic cells in the ganglion cell layer and decreased activation levels of caspase-8 and caspase-9. C5 plays a pivotal role in glaucoma pathogenesis; its deficiency mitigates glaucoma severity in DBA/2J mice [139], whilst intravitreal injection of C5 antibodies inhibits MAC formation, preserving retinal function and RGCs [140]. Knockout of C3 or CFB significantly protects RGC function and visual acuity in glaucoma model rats [65]; The anti-C1q monoclonal antibody ANX007 demonstrated favourable intraocular safety and target binding capacity in Phase I clinical trials [141]. These findings reflect different levels of evidence: preclinical versus early clinical studies. Vashishtha et al. identified associations between aqueous humour CFB and CFD levels and POAG, providing a rationale for alternative pathway-targeted therapies [142]. Complement-targeting therapies such as ANX007 may be applied as precision interventions for patients exhibiting early complement overactivation, particularly those with elevated C3a/C3 ratios or MAC deposition in the retina. Biomarker-guided patient selection could optimise therapeutic benefit and minimise interference with physiological synaptic pruning. Differences between animal and human complement dynamics, and potential interference with physiological synaptic pruning, must be carefully considered.

Table 3 summarizes immunomodulatory therapeutic strategies for glaucoma. It outlines diverse agents targeting innate immunity, adaptive immunity, complement and microglial pathways, along with experimental models, IOP regulation, RGC and axon survival effects, research status and inherent limitations.

## 5. Discussion

Fellman et al. reported a case of a 66-year-old female patient with NTG and rheumatoid arthritis. During treatment with methotrexate for rheumatoid arthritis, her serum immunoreactivity to retinal proteins decreased significantly. Concurrently, over a three-year treatment period, her visual field examination results showed an improving trend [143]. Notably, during a brief treatment interruption, new patchy haemorrhages appeared in both optic discs. This suggests that fluctuations in immune activity may correlate with optic disc vascular events. The researchers hypothesised that methotrexate, by inhibiting monocyte proliferation and antibody synthesis, may reduce autoantibody levels directed against retinal antigens. This mechanism could exert beneficial effects on glaucomatous optic neuropathy occurring against an immunologically abnormal background. While combined therapy may reduce secondary inflammatory or immune-mediated stress, its role as a causal intervention for primary glaucoma remains hypothetical. The strongest evidence for methotrexate combined with etanercept comes from rheumatoid arthritis [144]. Methotrexate may provide broad, upstream suppression of chronic immune activation and corticosteroid-sparing benefits, whereas etanercept may more specifically inhibit TNF-α-driven glial activation and secondary neurodegenerative injury. Therefore, methotrexate combined with etanercept may be most relevant for selected glaucoma subgroups with prominent intraocular inflammation, systemic autoimmune disease, or uveitic secondary glaucoma. However, this strategy should currently be regarded as a hypothesis-generating therapeutic concept rather than an established treatment for primary glaucoma, and future studies should define the target population, route of administration, therapeutic window, structural and functional neuroprotection endpoints, and long-term safety profile.

Many candidate immunomodulators for glaucoma must reach the retina, RGCs, and optic nerve head at effective concentrations while minimising systemic immunosuppression and ocular toxicity. Topical administration is convenient but limited by tear clearance, corneal/conjunctival barriers, and blood–ocular barriers, making it inadequate for large biologics or intracellular agents. Intravitreal injection bypasses these barriers but carries risks, including repeated procedure burden, transient IOP spikes, inflammation, infection, and short drug residence. Sustained and targeted delivery systems—such as biodegradable implants, microspheres, hydrogels, nanoparticles, liposomes, and drug-eluting inserts—can prolong tissue exposure, protect labile molecules, reduce dosing, and limit systemic effects. Periocular or suprachoroidal routes may further improve posterior-segment targeting, though distribution, safety, and long-term tolerability require evaluation. Future studies should integrate pharmacokinetics, tissue bioavailability, local toxicity, IOP effects, and systemic immune risks. Effective drug delivery is therefore a critical determinant of whether immunomodulation can serve as a true disease-modifying strategy in glaucoma. Preclinical and limited clinical evidence are promising, but longitudinal, subtype-specific, and mechanistically informed studies are essential before broad clinical translation.

Animal models consistently demonstrate the mechanistic plausibility of immune-mediated neurodegeneration, yet translation to human glaucoma remains limited by cohort size, heterogeneity, and stage-specific effects. For instance, transient T-cell infiltration and autoantibody production observed in IOP-elevation models may not universally represent human NTG pathophysiology, where IOP-independent mechanisms predominate. Similarly, TNF-α blockade (etanercept, adalimumab) and complement inhibition (CR2-Crry, ANX007) show robust neuroprotection in preclinical models, but clinical evidence remains preliminary, underscoring the need for biomarker-guided patient selection. To advance precision immunotherapy in glaucoma, we recommend the following mechanistically guided, subtype-specific approaches:

1. Biomarker-Guided Cohort Stratification

Innate immunity: Track microglial activation (Iba1/CD68) and astrocyte A1 markers in early POAG and NTG subgroups.

Adaptive immunity: Monitor HSP27/HSP60-specific T cells, β2-agAAb, and Treg/Th17 ratios, especially in patients with systemic autoimmune comorbidity or gut microbiota perturbations.

Complement pathway: Quantify C3a/C3 ratio, MAC deposition, and C1q/C3 expression longitudinally in intermediate-stage patients to identify maladaptive complement activation windows.

2. Stage-Specific Intervention Timing

Early-to-intermediate stages: prioritise interventions that normalise microglial and complement activity without compromising physiological synaptic pruning (e.g., targeted CR2-Crry or selective TLR4 modulation).

Advanced stages: focus on combinatorial neuroprotection, including anti-inflammatory modulation (TNF-α inhibitors, minocycline), adaptive immune regulation (COP-1, Treg enhancement), and neurotrophic support (BDNF, IGF-1 supplementation), tailored to biomarker-defined dysregulation.

3. Combination and Precision Strategies

Integrate systemic and local interventions, e.g., systemic TNF-α blockade for patients with autoimmune comorbidity combined with local complement inhibition or microglial modulators.

Consider ocular drug delivery challenges: sustained-release intravitreal formulations or AAV-mediated gene therapies targeting complement or Fas/FasL pathways may enhance retinal penetration while minimising systemic adverse effects.

4. Longitudinal Mechanistic Cohorts

Establish prospective, subtype-stratified cohorts tracking immune biomarkers alongside OCT-RNFL, visual field progression, and IOP dynamics. This will allow temporal correlation of immune activation with structural and functional outcomes and resolve current uncertainties about causality versus secondary responses.

In conclusion, a refined understanding of glaucoma as a context-dependent immune-amplified neurodegenerative disease enables actionable, precision-guided strategies. Subtype- and biomarker-based approaches provide a roadmap for future clinical translation, emphasising the need for mechanistically anchored, stage-specific interventions. This framework reconciles conflicting evidence and moves beyond generic calls for longitudinal studies, offering concrete pathways toward immunomodulatory therapy in glaucoma.

## Figures and Tables

**Table 1:** Summary of Clinical Immune Evidence in Glaucoma Patients: Key clinical studies reporting immune changes in NTG and POAG, including autoimmunity, autoantibodies, immune cell shifts, and complement activation.

Study Type	Core Immune Finding	Glaucoma Subtype	Key Limitation
Case–control	~30% NTG patients have systemic autoimmune comorbidity (RA common)	NTG	Small, heterogeneous, cannot infer causality
Retrospective cohort	POAG patients show higher autoimmune prevalence (RA, psoriasis, uveitis, vitiligo)	POAG	Retrospective, confounders, association only
Post-KPro cohort	Faster glaucoma progression in systemic autoimmune disease	NTG/POAG	Small, single-centre, stage & treatment bias
Cross-sectional	Elevated autoantibodies (HSP27, HSP60, CALD1, PGAM1, VDAC2, HSPD1); β2-agAAb 82%	POAG	Small, early-stage, cross-sectional, pathogenicity unclear
Flow cytometry	Altered T-cell subsets: ↓ CD4^+^CD25^+^FoxP3^+^ Tregs, ↓ Treg/Th ratios, ↑ CD4^+^ & myeloid cells	POAG	Small, single timepoint; clinical relevance uncertain
Retina/aqueous	Complement deposition ↑ (C1q, C3, MAC); CFH ↓; C3a/C3 ratio ↑	POAG/NTG	Small tissue; post-mortem/surgical; stage-dependent
Peripheral blood T-cell analysis	HSP27/HSP60-specific CD4^+^ T cells ↑ 5-fold; autoantibodies ↑; persistent infiltration	POAG/NTG	Small cohorts; driver vs. secondary effect unclear
Animal models	CD4^+^ T cells activate microglia (M1), cytokines ↑, and Fas-FasL RGC apoptosis	POAG/NTG	Animal-specific; limited human validation

Note: Upward arrow (↑) denotes an upward trend; downward arrow (↓) denotes a downward trend.

**Table 2:** Integrated Immune Mechanisms and Therapeutic Targets Across Glaucoma Progression: Summarises key innate, adaptive, and complement immune mechanisms in glaucoma and their therapeutic targets.

Immune Component	Key Mechanisms	Stage	Impact on RGCs	Therapeutic Targets
Microglia	IOP/PIEZO1 → activation, TNF-α, IL-1β, cGAS–STING	Early → chronic	Neuroinflammation, RGC apoptosis, and ECM remodelling	Etanercept, Adalimumab, Minocycline, Ibudilast, TAK-242, LGALS3, CX3CL1-CX3CR1, PLX5622
Astrocytes/Müller cells	A1 astrocytes, C3 upregulation, ATP → microglia, ECM remodeling	Early → ongoing	BRB disruption, inflammation, indirect RGC injury	Ibudilast, Minocycline, Endothelin axis (Bosentan, SPP1), Microglia-targeted therapies
TLR signaling	TLR2/3/4 → NF-κB → cytokines, TM fibrosis	Early	RGC apoptosis, IOP elevation	TLR4 inhibitors (TAK-242)
T cells (HSP-specific)	CD4^+^ T cells → IFN-γ → microglia, Fas-FasL apoptosis	Intermediate → chronic	Sustained neuroinflammation, RGC apoptosis	COP-1, Treg enhancement (rapamycin, adoptive Tregs), Fas/FasL modulation (ONL1204, sFasL)
B cells/Autoantibodies	Dysregulated HSP27/HSP60, β2-AAb	Intermediate	Modulate IOP, RGC stress response	Indirect via immunoregulation (Methotrexate + Etanercept)
Complement	C1q/C3/MAC, synapse tagging, glial activation	Early → advanced	Synapse loss, RGC apoptosis, amplified inflammation	CR2-Crry, CVF, ANX007, C5 antibodies, C3/CFB knockout

**Table 3:** Summary of Immunomodulatory Therapeutic Strategies in Glaucoma: This table summarises agents targeting innate and adaptive immunity, complement, and microglial pathways in glaucoma, including model systems, effects on IOP and RGC/axon survival, development stage, and limitations.

Strategy	Immune Target	Model System	Effect on IOP	Effect on RGC	Key Limitations
Etanercept	TNF-α inhibition	Rat glaucoma model	No effect	Preserves axons, maintains RGC density	IOP-independent; long-term safety and ocular delivery uncertain
Adalimumab	TNF-α/TNFR1/TNFR2 axis	Clinical (uveitis), preclinical glaucoma	No effect	Potential RGC protection	Direct evidence in primary glaucoma lacking; receptor-specific effects unknown
Bupropion	TNF suppression via cAMP	Retrospective clinical study	N/A	Reduced OAG risk (association only)	Causal mechanism unproven; not a validated therapy
Minocycline	Glial inhibition, anti-inflammatory	DBA/2J mouse, laser-induced rat	No effect	↑ RGC survival, ↓ microglial activation	Optimal dose/route unknown; retinal toxicity at high dose; limited in advanced disease
Caffeine/A_2_ₐR antagonists	Adenosine receptor pathway	Laser-induced OHT rat, ischemia-reperfusion rat	↓ IOP (OHT rats)	↓ RGC loss, ↓ microglial activation	Clinical applicability untested
FM101	A_3_AR modulator	Rat toxicity studies	N/A	↓ RGC loss	Translation to humans is ongoing
Ibudilast	PDE4 inhibition, glial modulation	Rat elevated IOP	No effect	Preserves RGCs & axons	Dosing, delivery, and long-term safety in humans are unknown
Bosentan	Endothelin receptor blockade	DBA/2J mouse	No effect	Mitigates RGC damage	The effect on IOP progression is limited; translation to humans is not fully validated
TAK-242 (Resatorvid)	TLR4 inhibition	Mouse optic nerve compression	N/A	↑ RGC survival	Timing- and model-dependent; clinical translation pending
LGALS3 inhibition/TD139	Microglial activation	DBA/2J, microsphere models	N/A	↑ RGC survival	Efficacy depends on disease type and timing
CX3CL1 (recombinant)	Microglial homeostasis (CX3CL1-CX3CR1 axis)	Rodent intravitreal injection	N/A	Neuroprotective	Optimal delivery and dosing undefined
PLX5622	CSF1R inhibitor, microglia depletion	Ischemia–reperfusion rat	N/A	↓ neuroinflammation	Ineffective in acute optic nerve injury; model- and timing-dependent
Cop-1 (Glatiramer acetate)	T cell modulation	Rat elevated IOP, clinical acute PACG	N/A	↑ RGC survival	Dose, timing, and combination therapy optimisation needed
Treg enhancement (rapamycin or adoptive transfer)	Regulatory T cells	Glaucoma models	N/A	Protects RGCs	Optimal induction and local delivery require validation
Fas/FasL modulation (ONL1204, sFasL AAV)	Fas receptor pathway	Glaucoma models	N/A	↓ RGC apoptosis, ↓ axon loss	Requires gene therapy delivery; timing-sensitive
Complement inhibition (CR2-Crry, CVF, anti-C1q/ANX007, C3/CFB knockout)	Complement cascade	Mouse glaucoma models	N/A	Preserves RGC soma and axons	Stage- and biomarker-dependent; potential interference with physiological pruning

Note: N/A indicates the relevant content was not mentioned in the included studies. ↑ means an increasing trend, whereas ↓ means a decreasing trend.

## Data Availability

No new data were created or analyzed in this study.
